# Reviewed article: Silva AGV, Miguez FGG, Siqueira LLC, Leite FMC. Perception of stress and its association with socioeconomic, behavioral and clinical characteristics of women: a cross-sectional study in Vitória, Espírito Santo. Epidemiol Serv Saude. 2025:34;e 20240224

**DOI:** 10.1590/S2237-96222025v34e20240224.b

**Published:** 2025-08-04

**Authors:** Oswaldo Villa

**Affiliations:** USP, Periodontics

**Table:** 

Rating	Excellent	Good	Average	Below average	Poor
Interest		✓			
Quality			✓		
Originality			✓		
Overall			✓		

**Table:** 

Questionnaire	Yes	No	Not applicable
Does the manuscript contain new and significant information to justify publication?	✓		
Does the Abstract (Summary) clearly and accurately describe the content of the article?		✓	
Is the problem significant and concisely stated?	✓		
Are the methods described comprehensively?	✓		
Are the interpretations and conclusions justified by the results?	✓		
Is adequate reference made to other work in the field?	✓		

## Manuscript Structure

Length of article is: Too long

Number of tables is: Adequate

Number of figures is: Adequate

Please state any conflict(s) of interest that you have in relation to the review of this paper (state “none” if this is not applicable): None

## Comments

“Minhas preocupações com o artigo são:

1)“Sugiro análise estatística adicional por terem empregado abordagem pouco adequada/usual ao tipo dedado/delineamento.”

2) os resultados tem excesso de texto recomenda-se estruturar melhor o texto, colocando texto e umatabela o ilustração em seguida, optar por linguaje simples e direta.

3)presenta interpretação de resultados que pertencente ao discussão.

4) a discussão presenta dados incompletos de estadística, precisa completar os dados e tem trechos quefalam de um estudo mas não especificam qual estudo.

5) Observar limite de palavras nas instruções aos autores: da introdução até o final da discussão, omanuscrito deve conter no máximo 3.500 palavras

6) As conclusões devem ser revisadas para garantir que sejam justificadas pelos resultados e respondamdiretamente à pergunta de pesquisa.”

7) “Evite conclusões exageradas ou que extrapolam os resultados.”

Apresente resultados mais completos e conclusivos, expandindo a análise e interpretação dos dados

8) As imagens, gráficos e figuras precisam ser de alta qualidade e adequadas para publicação.” “Assegure-sede que as ilustrações contribuam para a clareza e o impacto visual do trabalho.”

